# FGFR signaling maintains a drug persistent cell population following epithelial-mesenchymal transition

**DOI:** 10.18632/oncotarget.13117

**Published:** 2016-11-04

**Authors:** Wells S. Brown, Saeed Salehin Akhand, Michael K. Wendt

**Affiliations:** ^1^ Purdue University Center for Cancer Research, Department of Medicinal Chemistry and Molecular Pharmacology, Purdue University, West Lafayette, Indiana 47907, USA

**Keywords:** breast cancer, Her2 resistance, FGFR, EMT, covalent kinase inhibitor

## Abstract

An emerging characteristic of drug resistance in cancer is the induction of epithelial-mesenchymal transition (EMT). However, the mechanisms of EMT-mediated drug resistance remain poorly defined. Therefore, we conducted long-term treatments of human epidermal growth factor receptor-2 (Her2)-transformed breast cancer cells with either the EGFR/Her2 kinase inhibitor, Lapatinib or TGF-β, a known physiological inducer of EMT. Both of these treatment regimes resulted in robust EMT phenotypes, but upon withdrawal a subpopulation of TGF-β induced cells readily underwent mesenchymal-epithelial transition, where as Lapatinib-induced cells failed to reestablish an epithelial population. The mesenchymal population that remained following TGF-β stimulation and withdrawal was quickly selected for during subsequent Lapatinib treatment, manifesting in inherent drug resistance. The Nanostring cancer progression gene panel revealed a dramatic upregulation of fibroblast growth factor receptor 1 (FGFR1) and its cognate ligand FGF2 in both acquired and inherent resistance. Mechanistically, FGF:Erk1/2 signaling functions to stabilize the EMT transcription factor Twist and thus maintain the mesenchymal and drug resistant phenotype. Finally, Lapatinib resistant cells could be readily eliminated using recently characterized covalent inhibitors of FGFR. Overall our data demonstrate that next-generation targeting of FGFR can be used in combination with Her2-targeted therapies to overcome resistance in this breast cancer subtype.

## INTRODUCTION

Epithelial-mesenchymal transition (EMT) is a physiological process whereby epithelial cells breakdown cell-cell junctions and transiently or permanently transition into a state that is more representative of migratory cells [1]. Transient EMT occurs during developmental events (Type I) and during wound repair (Type II) [2]. In contrast, initiation of “Type III” or pathological EMT contributes to the invasion and ultimate metastasis of cancer cells [3, 4]. Physiologic and pathologic EMT can be induced by cytokines such as TGF-β and HGF [5]. Mechanistically, EMT is mediated through several signaling pathways that act in concert to modulate expression of “master” EMT transcription factors such as the basic helix loop helix (bHLH) factor Twist. Twist leads to direct and indirect downregulation of epithelial markers and upregulation of mesenchymal markers [6]. More recent findings demonstrate that EMT can also be directly initiated by treatment with kinase inhibitors and that this transition to a mesenchymal state facilitates tumor cell persistence in the presence of these molecularly-targeted compounds [7]. Despite these advances in elucidating the molecular players involved in the conversion of cells from an epithelial to mesenchymal state, little is known about the changes in molecular signaling pathways that result as a consequence of these differing EMT stimuli.

Human epidermal growth factor rector 2 (Her2) is member of the ErbB family of growth factor receptors and its expression is amplified in 20-25% of breast cancer patients [8]. Treatment options of Her2+ breast cancer patients has improved with the advent of targeted antibodies (Pertuzumab and Trastuzumab) and kinase inhibitors (Lapatinib, Afatinib, Neratinib), but inherent and acquired resistance to these therapies remains a major clinical problem for patients with this breast cancer subtype [9, 10]. While the morphologic induction of EMT is an underlying feature of resistance to these ErbB-targeting compounds a lack in understanding of the molecular mechanisms of this event has prevented the development of therapeutics capable of targeting this drug resistant state [11, 12].

Previous studies by our lab and others have demonstrated that expression of fibroblast growth factor receptor 1 (FGFR1) is dramatically increased during TGF-β-induced EMT and plays a critical role in metastatic tumor growth [13–15]. Along these lines, FGFR has previously been suggested as a mechanism of resistance to Her2 and other targeted molecular therapies [9, 16, 17]. Given these previous findings we sought to address the hypothesis that FGFR functions as a driver of EMT-associated drug resistance. Our results clearly demonstrate that following TGF-β-induced EMT Her2-transformed cells maintain a resident mesenchymal cell population that is highly resistant to ErbB inhibition. Conversely, Lapatinib-resistant cells become increasingly sensitive to our recently characterized covalent inhibitors of FGFR [15, 18, 19]. Overall, our data suggest that combination therapies utilizing both Her2 and FGFR inhibitors will result in more durable clinical responses in patients with this subtype of breast cancer.

## RESULTS

### Acquisition of resistance to Lapatinib results in a stable mesenchymal phenotype

**Figure 1:**
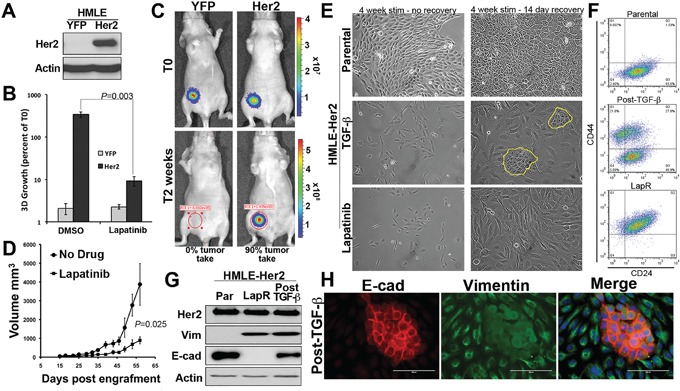
Acquisition of resistance to Lapatinib results in a stable mesenchymal phenotype **A.** Stable overexpression of Her2 in human mammary epithelial (HMLE) cells was verified by immunoblot. Actin served as a loading control. **B.** Firefly luciferase expressing control (YFP) and Her2 overexpressing HMLE cells were grown under 3D organotypic conditions in the presence or absence of Lapatinib (1 μM) for 11 days. 3D growth was quantified by bioluminescence and data is normalized to the plated values. **C.** Control (YFP) and Her2 overexpressing HMLE cells were engrafted onto the mammary fat pad of nu/nu mice. Mice were imaged 30 minutes after engraftment (T0) and two weeks later. **D.** Following fat pad engraftment (15 days) mice bearing Her2-transformed HMLE tumors (n = 5 mice per group) were treated with Lapatinib (50 mg/kg/48hours) via oral gavage. Mammary tumor size was quantified using digital calipers at the indicated time points resulting in the indicated P value. **E.** Her2-transformed HMLE cells were grown in the presence of TGF-β1 or Lapatinib for a period of 4 weeks as described in the materials and methods. The TGF-β1 and Lapatinib were subsequently withdrawn and these cells were cultured for an additional 14 days. The return of epithelial cells in cultures following TGF-β1 treatment is highlighted in yellow. **F-H.** Following the TGF-β and Lapatinib treatment and withdrawal protocols described in panel E these cultures were analyzed by flow cytometry for cell surface expression of CD44 and CD24 (F), immunoblot (G), or immunofluorescence (H) for E-cadherin (E-cad) and vimentin (Vim).

Acquisition and/or inherent resistance to Her2 targeted therapies can take place through a number of mechanisms that may be unique to the other underlying mutations that are specific to particular tumor models [20]. Therefore, in an effort to elucidate more global mediators of resistance to the clinically used EGFR/Her2 kinase inhibitor, Lapatinib, we utilized a model in which directed overexpression of Her2 mediates transformation of otherwise normal mammary epithelial cells [21]. As shown in Figure 1A Her2 was overexpressed in non-transformed human mammary epithelial cells (HMLE) (Figure 1A). Ectopic expression of Her2 allowed for culture of the HMLE cells in standard serum containing media (not shown), reduced expression of CD24, facilitated aberrant growth under 3D culture conditions and led to tumor formation in mice (Figure 1B and 1C, Supplementary Figure S1A, S1B and S1C). Importantly, Her2-driven cell growth in 3D culture and tumor growth *in vivo* could be significantly, but not completely, inhibited by treatment with Lapatinib (Figure 1B and 1D). Long-term culture (4 weeks) of Her2-transformed HMLE cells with regular addition of Lapatinib yielded a proliferative cell population that displayed a highly mesenchymal morphology (Figure 1E). A similar yet distinct cell morphology could also be elicited in these cells upon long-term culture with TGF-β1 (Figure 1E). Treatment of parental HMLE-Her2 cells with the covalent ErbB inhibitor Afatinib lead to a similar mesenchymal morphology but a proliferative population could not be established (Supplementary Figure S1B). Both TGF-β and Lapatinib-induced EMT events lead to the dramatic upregulation of CD44. However, upon withdrawal of these differential stimuli only those cells induced to undergo EMT by TGF-β reestablished an epithelial population where as a Lapatinib-induced EMT event was stably maintained following withdrawal of the drug (Figure 1E and 1F). The stable versus transient EMT events induced by Lapatinib and TGF-β respectively could further be visualized by immunoblot and immunofluorescence for the mesenchymal marker vimentin and the epithelial marker E-cadherin (Figure 1G, 1h and Supplementary Figure S1C). Overall, these data clearly establish the stable verses transient nature of EMT induced by EGFR/Her2 inhibition versus that induced by TGF-β. Furthermore, they demonstrate how TGF-β-induced EMT and mesenchymal-epithelial transition (MET) results in the formation of a heterogeneous cell population consisting of both epithelial and mesenchymal cells.

### TGF-β-induced EMT primes cells to be inherently drug resistance

**Figure 2:**
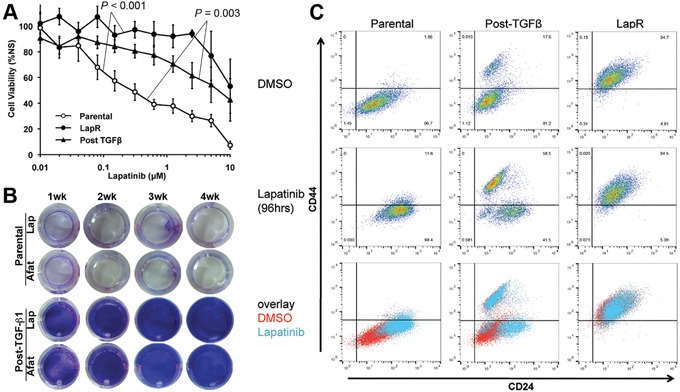
TGF-β-induced EMT primes cells to be inherently drug resistance **A.** Control (parental), Lapatinib Resistant (LapR) and TGF-β treated and withdrawn (Post–TGF-β) Her2-transformed HMLE cells were treated with the indicated concentrations of Lapatinib for 96 hours and subsequently assayed for cell viability. Data are normalized to the untreated parental cells and represent 3 independent experiments completed in triplicate resulting in the indicated P values. **B.** Confluent monolayers of control (parental) and post-TGF-β Her2-transformed HMLE cells were treated with ErbB inhibitors Lapatinib (Lap; 1μM) or Afatinib (Afat; 1μM) for the indicated amounts of time and surviving cells were stained with crystal violet. **C.** The indicated populations of cells were treated with vehicle (DMSO) or Lapatinib (1μM) for 96 hours and subsequently analyzed by flow cytometry for cell surface expression of CD44 and CD24.

Given the similarities between cell populations that could be generated by TGF-β and Lapatinib induced EMT we next sought to investigate the ability of TGF-β-induced EMT to generate drug resistant cells. Therefore, we utilized cell viability assays to quantify the differential response of parental Her2-transformed HMLE cells as compared to cells that had been treated (4 weeks) and removed (4 weeks) from either Lapatinib or TGF-β. Indeed, consistent with their maintenance of a CD44^high^ mesenchymal phenotype Lapatinib selected cells remained highly resistant even after prolonged culture in the absence of drug (Figure 2A). Cell viability assays also established that Lapatinib resistant cells are similarly more resistant to covalent pan-ErbB inhibitor Afatinib (Supplementary Figure S1D). Surprisingly, a post-TGF-β cell population was also highly resistant to Lapatinib treatment even though these cells had not previously been treated with this compound (Figure 2A). Moreover, while a 4-week treatment with 1 μM Lapatinib or Afatinib results in the sparse persistence of very few parental cells those cells that had been pretreated with TGF-β are stably resistant and proliferative in the presence of these drug treatments (Figure 2B). Flow cytometry for CD44 and CD24 demonstrated that treatment of the heterogeneous post-TGF-β cell population leads to a robust selection for the CD44^high^ population (Figure 2C; middle column). This treatment strategy had no affect on the Lapatinib resistant cells (Figure 2C; right column). Overall, these data clearly demonstrate that the CD44^high^ mesenchymal population that remains following TGF-β-induced EMT:MET possess an inherent resistance to ErbB inhibition.

### Her2 inhibition only targets a CD44^low^/epithelial cell population

**Figure 3:**
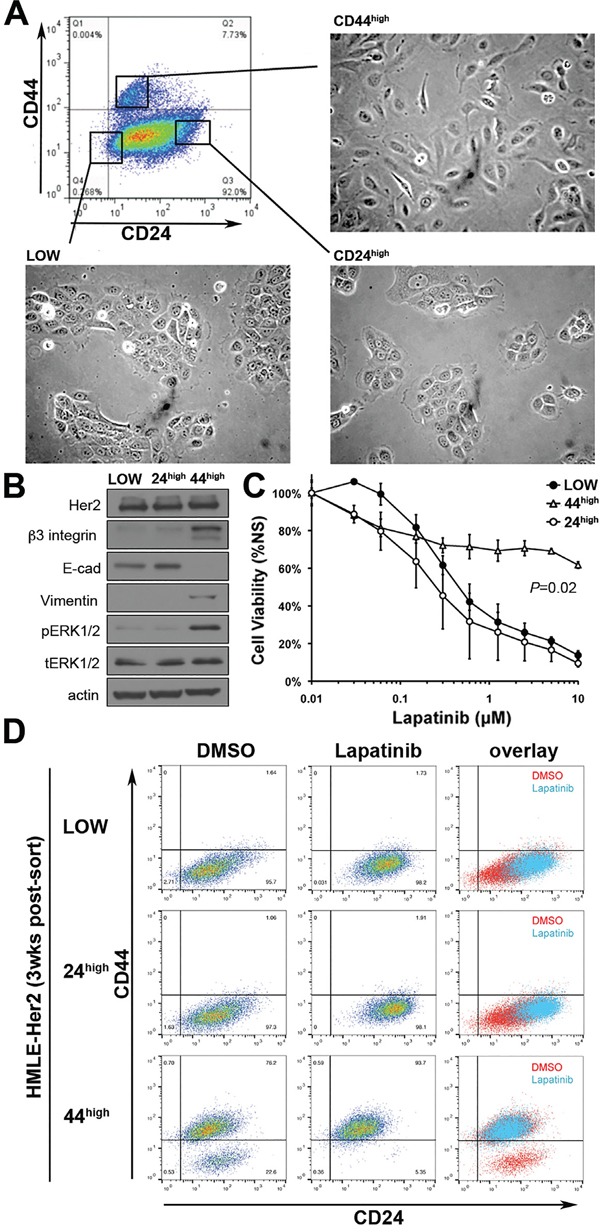
Her2 inhibition only targets a CD44^low^/epithelial cell population **A.** The indicated populations of post-TGF-β treated Her2-transformed HMLE cells were separated by FACS. The epithelial versus mesenchymal morphology of each population is shown. **B.** The expression levels of Her2, β3 integrin, E-cadherin (E-cad) and Vimentin for each of the FACS sorted populations were determined by immunoblot. The phosphorylation of Erk1/2 was also determined. Actin and total Erk1/2 served as loading controls. **C.** The populations isolated in panel A were subjected to a 96-hour treatment with the indicated concentrations of Lapatinib and cell viability was assayed. Data are normalized to untreated values and are the mean ±SE of three independent experiments completed in triplicate resulting in the indicated *P* values **D.** Three weeks after FACS these populations were again assayed for cell surface expression of CD44 and CD24 under vehicle treated (DMSO) or 96 hour Lapatinib-treated (1μM) conditions.

To further confirm our observations from Figures 1 and 2 we utilized fluorescence activated cell sorting (FACS) to separate cells that had been treated and withdrawn from TGF-β based on cell surface expression levels of CD44 and CD24 (Figure 3A). Expression of Her2 was equal between these isolated populations as determined by western blot, but the CD44^high^/mesenchymal population displayed enhanced basal phosphorylation of Erk1/2 (Figure 3B). More importantly, cell viability assays confirmed that only the CD44^high^ population was inherently resistant to Lapatinib (Figure 3C). Both CD44^low^ populations displayed an IC50 to Lapatinib that was similar to parental cells even though these cell populations were derived from the same TGF-β treated culture as the CD44^high^ cells (Figure 3C). We next sought to determine the repopulating ability of these post-TGF-β isolated subpopulations and found that after three weeks of passage subsequent to FACS isolation, only the CD44^high^ population now contained both epithelial and mesenchymal populations (Figure 3D; bottom row). Importantly, the CD44^low^ population that emerged following FACS isolation of CD44^high^ cells could be eliminated by treatment with Lapatinib (Figure 3D). Together with the data from Figure 2 these findings indicate that following EMT and MET cells regain their sensitivity to Lapatinib, but the resident population of CD44^high^ mesenchymal cells can manifest inherent resistant to ErbB inhibition.

### FGFR signaling is sufficient to elicit resistance to ErbB inhibition

**Figure 4:**
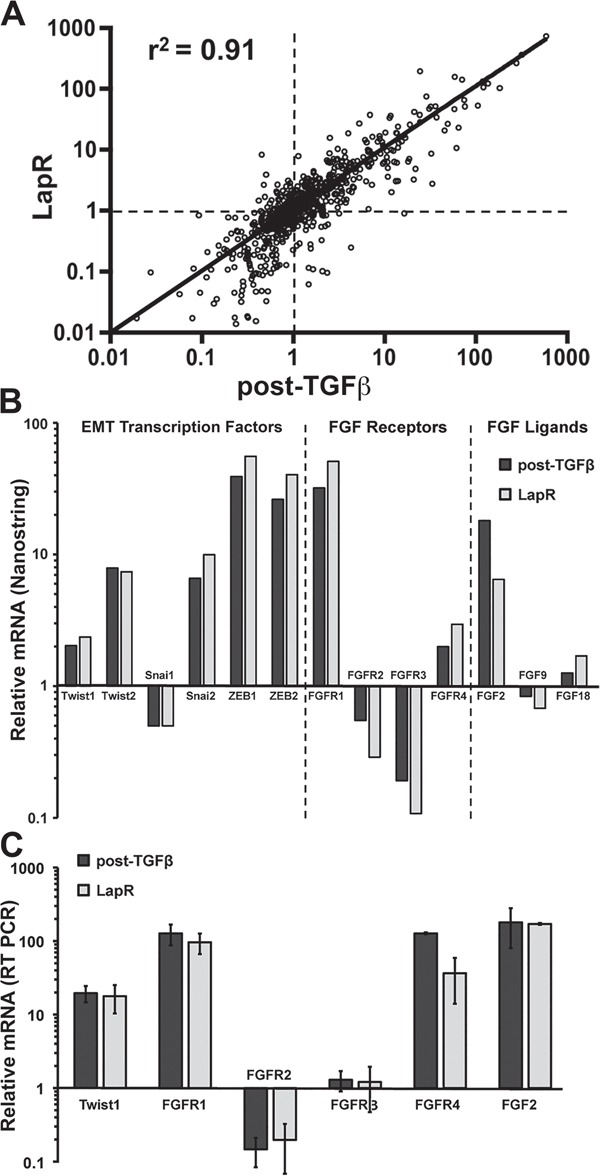
FGFR signaling is similarly increased in acquired and inherent drug resistance **A.** Gene expression was analyzed in parental Her2-transformed HMLE cells, the post-TGF-β cell population, and Lapatinib resistant (LapR) cells using the Nanostring PanCancer Progression panel. Plot shows the fold change in expression of the 770 genes in the panel for the post-TGF-β cells (X-value) and LapR cells (Y-value) normalized to parental values as 1 (dashed lines). This comparison resulted in the indicated coefficient of determination (r^2^). **B.** Selected data from the Nanostring panel showing similar gene expression changes in the indicated EMT transcription factors, FGF receptors and FGF ligands. **C.** Expression changes in these factors were confirmed by RT-PCR analysis. Data are normalized to parental HMLE-Her2 values and are the mean ± the SD of three independent samples.

**Figure 5:**
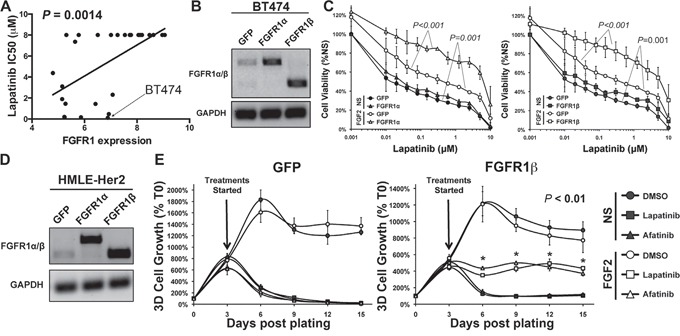
FGFR1:FGF2 signaling is sufficient to drive resistance to ErbB inhibition **A.** CCLE data comparing FGFR1 expression levels to the IC50 values for Lapatinib. The Lapatinib sensitive BT474 cells are indicated. **B.** RT-PCR analyses verifying stable overexpression of the α and β isoforms of FGFR1 in the BT474 cells. GAPDH served as a loading control. **C.** BT474 cells as shown in panel B were treated with Lapatinib at the indicated concentrations for 96 hours in the presence or absence of exogenous FGF2 (20 ng/ml). Subsequent to this treatment cell viability was assessed. Data are normalized to untreated GFP control cells and are the mean ± SD of two independent experiments completed in triplicate. **D.** RT-PCR analyses verifying stable overexpression of the α and β isoforms of FGFR1 in the HMLE-Her2 cells. GAPDH served as a loading control. **E.** The HMLE-Her2 cells shown in panel D were plated under 3D organotypic conditions in the presence or absence of Lapatinib (1 μM), Afatinib (100 nM), and FGF2 (20 ng/ml) and longitudinal 3D cell growth was quantified at the indicated time points via bioluminescence. Data are normalized to the plated values and are the mean ± SE of two independent experiments completed in triplicate resulting in the indicated P values.

We next sought to characterize the CD44^high^/mesenchymal cell population produced following acquired resistance to Lapatinib (LapR) as compared to those cells displaying inherent Lapatinib resistance generated via TGF-β stimulation. To do this we utilized the Nanostring Pancancer, cancer progression gene expression panel. This panel consists of 770 genes known to be associated with angiogenesis, EMT, extracellular matrix remodeling, and metastasis. Analysis of the LapR cells and the post-TGF-β cells as compared to their parental HMLE-Her2 counterparts revealed robust upregulation of several extracellular matrix proteins, integrins and EMT transcription factors (Figure 4A and 4B and Supplementary Table S1). Importantly, FGFR1 was the most upregulated growth factor receptor in post-TGF-β and LapR cells and PCR analyses specified this increase to be the –iiic isoform of FGFR1 and confirmed increased expression of its cognate ligand FGF2 (Figure 4C and Supplementary Figure S2A and S2B). To further confirm the ability of FGFR signaling to drive resistance to ErbB inhibition we analyzed the CCLE database by comparing the documented IC50 values for Lapatinib and the corresponding expression levels of the FGFR1 in 27 analyzed breast cancer cell lines (Supplementary Figure S3A). This comparison demonstrated a highly significant positive correlation between FGFR1 expression and the IC50 value for Lapatinib across these different cell lines (Figure 5A). Furthermore, directed overexpression of both the full length (α) or truncated (β) –iiic isoforms of FGFR1 in the Lapatinib sensitive BT474 cells led a highly significant resistance to Lapatinib (Figure 5B and 5C, Supplementary Figure S3B). Finally, similar overexpression of FGFR1 in our HMLE-Her2 cells also supported significant resistance to Lapatinib and Afatinib in a 3D organotypic culture (Figure 5D and 5E, Supplementary Figure S3C). Importantly, in both of these systems FGFR1-mediated resistance to ErbB inhibition required the addition of exogenous FGF2 (Figure 5C and 5E). These data clearly demonstrate that FGF2:FGFR1 signaling is sufficient to facilitate resistance to ErbB inhibition.

### An FGFR:Erk1/2:Twist positive feedback loop stabilizes a CD44^high^, drug resistant phenotype

**Figure 6:**
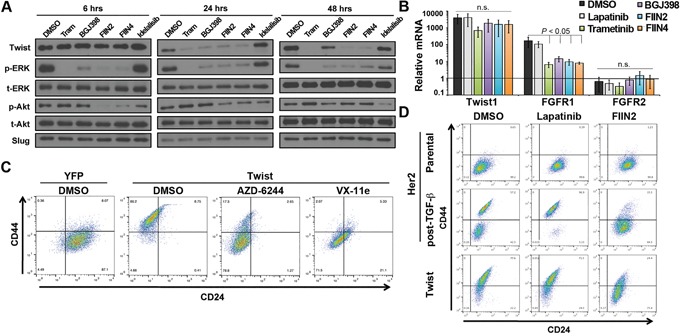
An FGFR:Erk1/2:Twist positive feedback loop stabilizes a CD44^high^, drug resistant phenotype **A.** HMLE cells constructed to overexpress Twist were treated with a MEK inhibitor Trametinib (Tram), FGFR inhibitors (BGJ-398, FIIN2, or FIIN4) or a PI3K inhibitor (Idelalisib) for the indicated amounts of time. These cells were subsequently analyzed for phosphorylation of Erk1/2 and Akt and total Erk1/2 and Akt served as loading controls. Expression of the EMT transcription factors Twist and Slug were also assessed. **B.** Levels of Twist, FGFR1 and FGFR2 mRNA were assessed by RT-PCR following a 48 hour treatment with 100 nM Trametinib or 1 μM of the other indicated inhibitors. Data are normalized to expression of levels of each gene found with control (YFP expressing) HMLE cells and are the mean ±SD of three independent experiments. **C.** Control (YFP) and Twist expressing HMLE cells were assayed by flow cytometry for cell surface expression of CD44 and CD24. Where indicated Twist expressing cells were treated with the MEK inhibitor (AZD-6244) or the Erk inhibitor (Vx-11e) for 96 hours prior to analysis. **D.** Her2 transformed HMLE cells were left untreated (parental) or were treated and allowed to recover from TGF-β1-induced EMT (post-TGF; as described in Figure 1). These cell populations were subsequently treated with Lapatinib or FIIN2 for 96 hours and analyzed by flow cytometry for cell surface expression of CD44 and CD24. Twist expressing HMLE cells were similarly treated and analyzed.

Overexpression of the bHLH EMT transcription factor Twist in the HMLE cells leads to a robust induction of a CD44^high^ mesenchymal phenotype [21]. Consistent with our previous report using the normal murine mammary gland cell model, we found that Twist driven EMT includes a marked upregulation of FGFR1-β-iiic in the HMLE cells (Supplementary Figure S2A, [15]). MAPK phosphorylation is required to stabilize Twist and prevent its proteosome-mediated degradation [22, 23]. Accordingly, we observed reversible loss of Twist protein, but not mRNA expression in the murine model using the direct Erk2 inhibitor VX11E (Supplementary Figure S4). Additionally, treatment of the HMLE cells with the MEK1/2 inhibitors Trametinib or AZD6244 resulted in a dose dependent loss of recombinant Twist protein that mirrored inhibition of Erk1/2 phosphorylation (Supplementary Figure S5A-S5B). Moving upstream, we observed this same loss in Twist protein, but not mRNA could be elicited upon treatment with reversible and covalent FGFR inhibitors (Figure 6A and 6B). Illustrating the importance of covalent FGFR inhibition, Twist protein levels returned 48 hours after addition of BGJ-398, but remained diminished with the irreversible compounds (Figure 6A, right panel). As a result of this loss in Twist protein FGFR1 mRNA levels were significantly decreased following FGFR and MEK inhibitor treatment while there was no effect on FGFR2 mRNA (Figure 6B). As expected, we found expression of Twist induced a CD44^high^/CD24^low^ phenotype as compared to control HMLE cells. However, treatment with MEK/ERK inhibitors partially reversed this phenotype as noted by decreasing expression of CD44 (Figure 6C). Similarly, covalent inhibition of FGFR also decreased CD44 expression in the HMLE-Twist cells (Figure 6D and Supplementary Figure S5C). Finally, covalent inhibition of FGFR eliminated the CD44^high^ post-TGF-β population while sparing the CD44^low^ cells (Figure 6D and Supplementary Figure S5D). Overall, these data indicate that maintenance of a Lapatinib resistant CD44^high^ phenotype is dependent on FGFR-driven activation of MEK/ERK signaling, leading to stabilization of Twist.

### Combination of ErbB and FGFR therapy eradicates CD44^high^ and CD44^low^ cell populations

**Figure 7:**
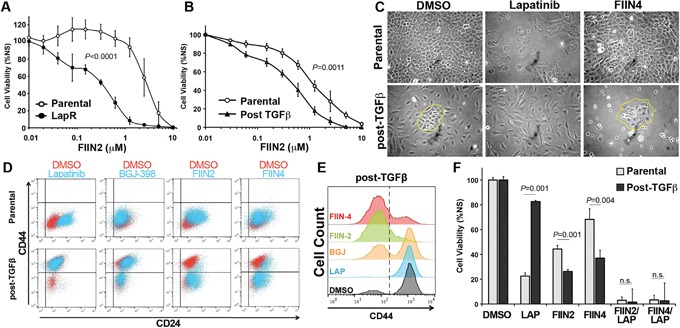
Combination of ErbB and FGFR therapy eradicates CD44^high^ and CD44^low^ cell populations **A.** Her2 transformed Parental and Lapatinib resistant (LapR) HMLE cells were treated with the FGFR inhibitor FIIN2 for 48 hours and assayed for cell viability. **B.** Her2 transformed Parental and TGF-β treated and recovered cells (post-TGFβ) HMLE cells were treated with the FGFR inhibitor FIIN2 for 96 hours and assayed for cell viability. Data in panels A and B are normalized to untreated cells and are the mean ± SE of at least two independent experiments completed in triplicate resulting in the indicated *P* values. **C.** Photomicrographs of cells described in panel B following the indicated ErbB (Lapatinib) or FGFR (FIIN4) inhibitor treatments. **D-E.** Cells were treated with the indicated inhibitors for 96 hours and subsequently analyzed by flow cytometry for cell surface expression of CD44 and CD24. **F.** Her2 transformed Parental and TGF-β treated and recovered (post-TGFβ) HMLE cells were treated with Lapatinib (LAP; 1 μM), FIIN2 (100 nM), FIIN4 (100 nM), or combinations of these compounds for 96 hours and assayed for cell viability. Data are normalized to untreated cells and are the mean ± SE of at three independent experiments completed in triplicate resulting in the indicated *P* values.

Given the differential sensitivity of post-TGF-β CD44^low^ and CD44^high^ cells to Lapatinib and FIIN4, respectively we next sought to evaluate the sensitivity of the LapR cells to covalent FGFR inhibition. HMLE-Her2 cells that spontaneously acquired resistance to Lapatinib were significantly more sensitive to covalent FGFR inhibition as compared to their parental counterparts (Figure 7A). Similarly, the heterogeneous post-TGF-β cell population was also significantly more sensitive to FIIN2 and FIIN4 as compared to the HMLE-Her2 parental cells (Figure 7B and Supplementary Figure S6). Upon closer examination of the post-TGF-β cultures we could readily visualize the epithelial versus mesenchymal cell selection that took place upon Lapatinib or FIIN4 treatment respectively (Figure 7C). This cell type specific selection could also be detected using flow cytometery for CD44/CD24 (Figure 7D and 7E). Importantly, these studies again demonstrated the inability of an ATP-competitive FGFR inhibitor to eliminate the CD44^high^, Lapatinib resistant cells (Figure 7D and 7E). Finally, cell viability assays clearly demonstrate that combined treatment of Lapatinib with either FIIN2 or FIIN4 eradicates cells capable of resisting single agent treatment (Figure 7F).

## DISCUSSION

Using an *in vivo* reporter for E-cadherin we recently established that as breast cancer cells disseminate they undergo a robust EMT followed by a partial MET during metastatic outgrowth [14]. Our data herein using single cell flow cytometric analyses suggest that this partial MET actually results from the creation of a heterogeneous epithelial and mesenchymal metastatic tumor cell population. These data demonstrate a merger in the ideas between cellular plasticity and tumor heterogeneity, two important concepts in EMT:MET biology. These findings further suggest that the physiological processes of EMT engender metastatic tumors with a resident mesenchymal cell population that is inherently resistant to currently used targeted therapies. Herein we utilized the Her2-driven HMLE model to sequentially demonstrate diminution of CD24 by directed Her2 overexpression and increased CD44 expression following induction of EMT. Subsequent to TGF-β-induced EMT:MET treatment with Lapatinib or the more recently developed covalent pan-ErbB inhibitor Afatinib results in immediate selection of the CD44^high^ mesenchymal cell population and inherent drug resistance. These data are significant because prior to TGF-β treatment we were unable to isolate a population of cells that spontaneously acquired resistance to Afatinib. Overall, these data are supported by clinical findings showing a CD44^high^/CD24^low^ phenotype is an independent prognostic factor for decreased Her2^+^ patient survival [24]. Furthermore, our data illustrate how tumors that have undergone EMT:MET have a higher degree of cellular heterogeneity and are therefore better poised to resist single agent pharmacological insults.

An important aspect of the current study has been the development of paired models of reversible (TGF-β-induced) and nonreversible (Lapatinib induced) EMT. Our findings therefore establish a unique platform in which to elucidate the factors that are required for mesenchymal cells to maintain a state of plasticity and transition back to an epithelial state. Ongoing studies in the lab are using numerous methods to characterize the plasticity of the post-TGF-β CD44^high^ cell population as compared to the stable mesenchymal phenotype that is generated upon spontaneous acquisition of Lapatinib resistance. Further understanding of the epigenetic factors involved in this plasticity could lead to novel pharmacological approaches to control epithelial-mesenchymal plasticity and thus modulate response to particular therapeutics [25].

Importantly, our findings clearly point to the critical role of FGFR in maintaining a mesenchymal and anti-ErbB resistance phenotype. Recent studies using the SKBR3 model of Her2 breast cancer suggest that FGFR is part of a more general kinome reprograming required for Lapatinib resistance [11]. However, the role of FGFR may have been underestimated in these studies due to the use of Type I ATP competitive FGFR kinase inhibitors. Indeed, the findings herein and our recently published studies clearly point to the enhanced efficacy of covalent FGFR inhibitors as compared to ATP competitive molecules [15]. Furthermore, our utilization of both the HMLE and BT474 models of Her2+ breast cancer clearly demonstrate that enhanced FGFR1 expression when in the presence of FGF ligand is sufficient to facilitate resistance to Lapatinib treatment.

**Figure 8:**
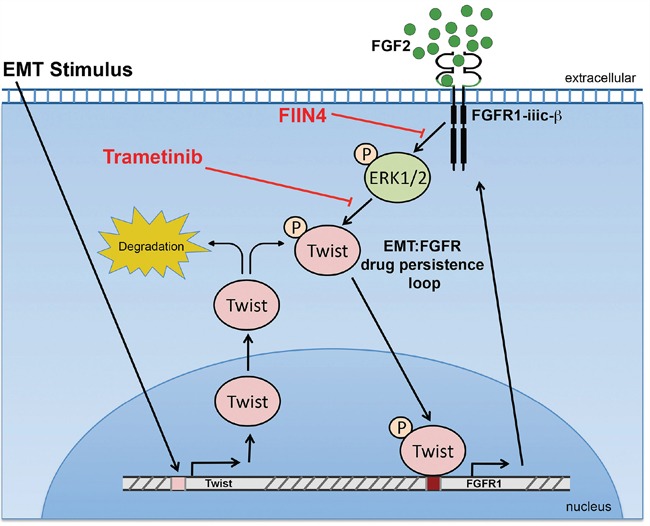
A Schematic representation of how FGFR signaling is bolstered following induction of EMT The activity of Erk1/2 stabilizes the expression of Twist, which drives the expression of FGFR1. In the continued presence of FGF ligand this constitutes a positive feedback loop that supports a mesenchymal population of cells that are resistant to ErbB-targeted agents.

Our recent studies in normal murine mammary gland cells establish that Twist is capable of inducing FGFR1 expression, results that are completely consistent with our data here using the HMLE model [15]. We further expand upon this mechanism by demonstrating a positive feedback loop in which FGFR signaling stabilizes Twist protein levels to maintain a mesenchymal phenotype (Figure 8). In addition to the regulation of the receptor we also find that FGF2 ligand was upregulated in our acquired and inherent Lapatinib resistant mesenchymal populations. These data are supported by previous findings that demonstrate an FGF2 signaling loop is at play in basal-like breast cancer [26]. Furthermore, together with our recent study we conclude that FGF2 and β3 integrin are part of an EMT signature that contribute to FGFR1-mediated drug resistance and metastatic progression [15]. Along these lines, recent clinical data indicate that unlike the autoactivation of Her2 upon gene amplification, FGFR1 amplification alone is insufficient to predict patient response to FGFR inhibitor therapy [27]. Mechanistically, we do not observe FGF stimulation alone to induce EMT (data not shown), but instead our findings suggest that FGFR acts to maintain Twist stability and thus prolongs a mesenchymal phenotype following EMT induction by alternate factors. Importantly, depletion of Twist is sustained for up to 48 hours using covalent FGFR inhibitors, but Twist quickly returns upon treatment with the ATP competitive compound, BGJ-398. Pharmacologic inhibition of FGFR, MEK and Erk support the notion that FGFR-mediated stabilization of Twist is mediated through an Erk-dependent mechanism (Figure 8). However, our studies have not completely ruled out other signaling mechanisms downstream of FGFR that may also be contributing to maintenance of a mesenchymal cell population. In any event, this sustained depletion of Twist seems to be critical as covalent inhibition of FGFR was required for successful elimination for Lapatinib-resistant mesenchymal cells.

Overall, our data herein demonstrate that the processes of EMT:MET plasticity enhance intra-tumor heterogeneity to support drug resistance. Furthermore, we demonstrate a mechanism whereby FGFR signaling maintains a drug persistent mesenchymal cell state. These studies indicate that combination of next generation ErbB inhibitors with FIIN4 will serve as an effective therapeutic strategy to prevent and/or reverse inherent or acquired resistance to currently used ErbB-targeting strategies. On going studies in the lab are taking a comprehensive approach to optimize the *in vivo* pharmacology for this combinatorial approach using a host of Her2-driven cell lines and patient-derived xenograft model systems. These studies have a high potential to translate into improved clinical strategies for treating patients with this subtype of breast cancer.

## MATERIALS AND METHODS

### Cell culture and reagents

Human mammary epithelial cells (HMLE) were obtained from Sendurai A. Mani (MD Anderson Cancer Center, Houston TX) and NMuMG and BT474 cells were purchased from the ATCC (Manassas, VA, USA). The HMLE cells were cultured in DMEM:F12 supplemented with insulin (10 μg/ml), EGF (10 ng/ml), and hydrocortisone (250 μg/ml), this media was mixed 1:1 with Mammary Epithelial Cell Growth Medium (MEGM) purchased from Lonza (Allendale, NJ, USA). Bioluminescent HMLE cells were engineered to stably express firefly luciferase via lentiviral transduction under the selection of Blasticidin. Her2 and Twist expressing HMLE cells and Twist expressing NMuMG cells were constructed via stable transduction using pBabe viral particles and selected for using puromycin. NMuMG, Her2 transformed HMLE and BT474 cells were cultured in DMEM supplemented with 10% FBS, 1% Pen/Strep, and 10 μg/mL of insulin. Plasmids encoding eGFP, FGFR1-α-IIIc (NM_023110.2) or FGFR1-β-IIIc (NM_023105.2) were purchased from Cyagen Biosciences (Santa Clara, CA, USA). These constructs were used to construct lentiviral particles, and stable transduction was selected for under Hygromycin selection. TGF-β1 and basic FGF (FGF2) were purchased from R&D systems (Minneapolis, MN). BGJ-398, Lapatinib, Afatinib, Trametinib, VX11e, AZD-6244, and Idelalisib were purchased from Selleckchem (Houston, TX), solubilized in DMSO and used at the indicated concentrations. FIIN-2 and FIIN-4 were synthesized as previously described and similarly solubilized in DMSO [15, 18].

### mRNA analyses

An RNA isolation kit from Omega bio-tek (Norcross, GA) was used to isolate RNA. These RNAs were analyzed by NanoString Technologies (Seattle, WA) using their PanCancer Progression Panel. In other assays RNA was reverse-transcribed using a cDNA synthesis kit from Thermo Fisher (Waltham, MA). Where indicated these cDNA libraries were assessed by standard PCR and analyzed by gel electrophoresis or by real-time PCR using iQ-SBYR green from BioRad (Hercules, CA). All oligonucleotides used are listed in Supplementary Table S2.

### Immuno assays

For immunoblot analyses cells were lysed using a modified RIPA lysis buffer containing 50 mM Tris, 150mM NaCl, 0.25% Sodium Deoxycholate, 1.0% NP40 and 0.1% SDS. This buffer was further supplemented with protease inhibitor cocktail, 10mM activated sodium ortho-vanadate, 40 mM β-glycerolphosphate and 20mM sodium fluoride. For immunofluorescence cells were fixed in 4% paraformaldehyde (PFA), permeablized in 0.1% triton-X 100 and processed using the indicated antibodies and appropriate secondary antibodies. For flow cytometry cells were fixed in 1% PFA, blocked in 1.0% bovine serum albumin and stained with the indicated antibodies that were directly conjugated to the fluorescent probes. All antibodies and their respective applications and concentrations are listed in Supplementary Table S3.

### Cell biological assays

Changes in cell viability were assayed using the CellTiter-Glo assay from Promega (Madison, WI). Where indicated bioluminescent HMLE-Her2 cells were grown under 3D culture conditions and cell growth was longitudinally tracked using a cell permeable luciferin (GoldBio, St. Louis, MO). Briefly, 2000 cells were plated in each well of a 96 well dish on top of a solidified 50 μl bed of basement membrane extract (BME) from Trevigen (Gaithersburg, MD). These cells were suspended in growth media containing DMEM, 10% FBS and 5% of the BME.

### Statistical analyses

Statistical values were defined using an unpaired Student's T-test, where a *P* value < 0.05 was considered significant. *P* values for all experiments are indicated.

## SUPPLEMENTARY FIGURES AND TABLES




