# Supplementary material for: FGFR signaling maintains a drug persistent cell population following epithelial-mesenchymal transition
Source: Oncotarget. 2016 Nov 4;7(50):83424–36. doi: 10.18632/oncotarget.13117 (PMC5347779; doi:10.18632/oncotarget.13117)
Supplement: Supplementary file 1 [file oncotarget-07-83424-s001.pdf]

FGFR signaling maintains a drug persistent cell population following epithelial-mesenchymal transition

SUPPLEMENTARY FIGURES AND TABLES

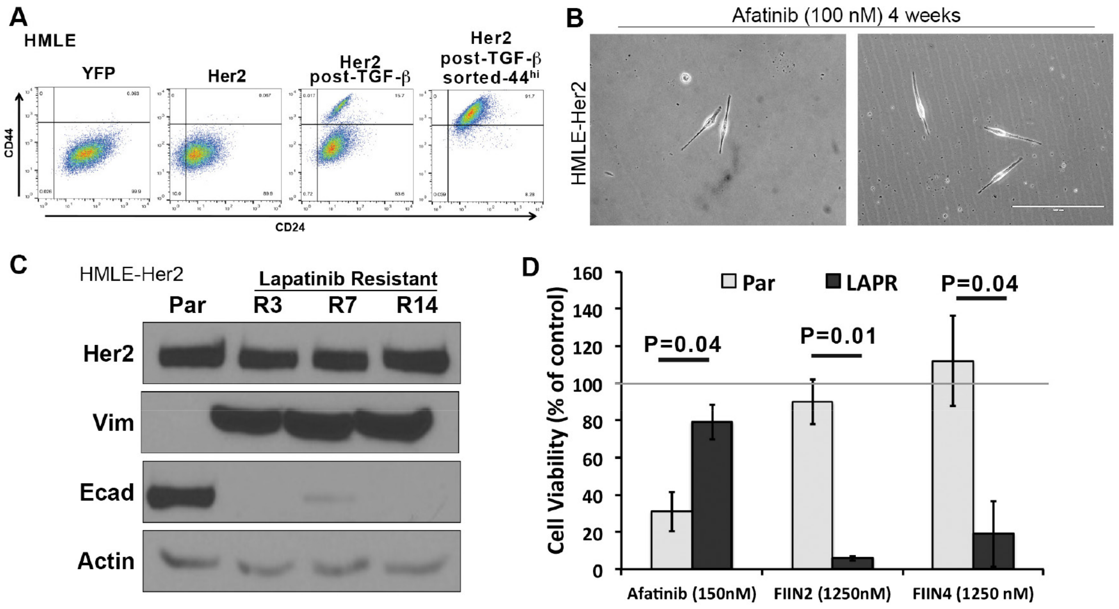

Supplementary Figure S1: Primary resistance to Lapatinib facilitates secondary resistance to Afatinib. A. HMLE cells stably expressing Her2 or YFP as a control were analyzed for cell surface expression of CD44 and CD24 by flow cytometry. Her2 expressing HMLE cells were treated with TGF-β for 4 weeks and subsequently allowed to recover in the absence of exogenous TGF-β for additional 4 weeks. The $CD44^{hi}$ expressing cells from this population were isolated via FACS. **B.** HMLE-Her2 cells were treated with the covalent pan-ErbB inhibitor Afatinib (1 µM) for 4 weeks and surviving mesenchymal cells could be observed but these cells never proliferated into a viable culture, even after removal of Afatinib. **C.** Three independent Lapatinib resistant cultures were analyzed by immunoblot for expression of Her2, E-cadherin (Ecad) and Vimentin (Vim). Actin served as a loading control. **D.** Parental, Her2 transformed HMLE cells (Par) and their Lapatinib resistant counterparts (LAPR) were treated with the indicated concentrations of Afatinib or the covalent FGFR inhibitors (FIIN2, FIIN4) for 48 hours and subsequently assessed for cell viability. Data are normalized to untreated cells set to 100% (indicated by the horizontal line) and are the mean ± SD of three independent experiments completed in triplicate resulting in the indicated *P* values.

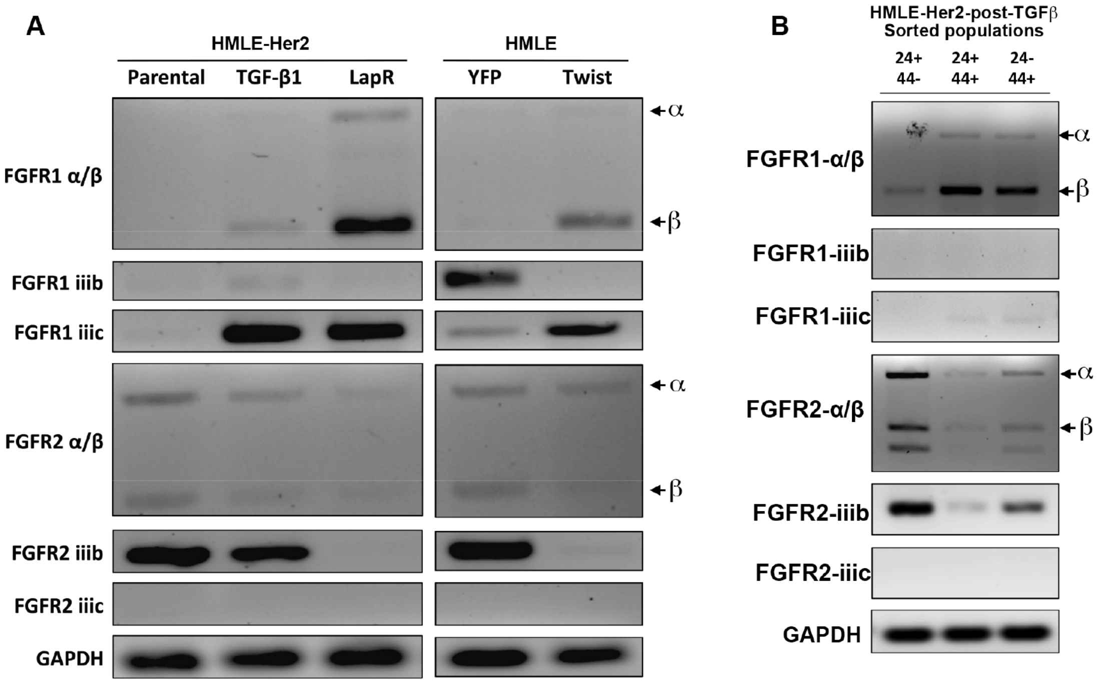

Supplementary Figure S2: Upregulation of the FGFR1-iiic-β isoform predominates drug-induced, TGF-β-induced and Twist-induced EMT events. A. Parental, post-TGF-β and Lapatinib resistant (LapR) Her2 transformed HMLE cells were analyzed by RT-PCR for inclusion of the –iiib or –iiic exon, which encode differential ligand binding domains. These cells were also analyzed for inclusion (α) or exclusion (β) of the third exon, which encodes the outermost Ig domain of these FGFR receptors. The sequences of these primer sets are listed in the Supplemental Table S2. **B.** Similar analyses were performed on the Her2-transformed post-TGF-β treated cells that were sorted by FACS based their expression levels of CD44 or CD24. In all cases GAPDH served as a loading control.

A

Primary Cell Line Name	FGFR1 mRNA Expression	ErbB2 mRNA Expression	Lapatinib IC50 (uM)
BT-474	6.886	11.753	0.116
ZR-75-30	5.864	12.944	0.167
AU565	5.242	12.997	0.183
SK-BR-3	5.231	11.993	0.196
MDA-MB-175-VII	6.926	ND	0.469
MDA-MB-453	6.417	10.005	1.438
HCC1954	5.276	12.629	1.458
CAL-85-1	5.149	7.827	1.900
MDA-MB-468	7.070	6.451	2.313
HDQ-P1	5.152	ND	3.105
HCC1806	4.796	7.352	4.379
HCC70	5.642	7.499	7.363

Primary Cell Line Name	FGFR1 mRNA Expression	ErbB2 mRNA Expression	Lapatinib IC50 (uM)
BT-20	5.307	8.032	8.000
BT-549	8.966	6.603	8.000
CAMA-1	7.538	7.851	8.000
EFM-19	5.777	8.968	8.000
HCC1187	7.768	7.995	8.000
HCC1395	7.725	6.233	8.000
HCC1569	7.009	12.101	8.000
HMC-1-8	7.257	6.364	8.000
Hs 578T	9.056	6.990	8.000
Hs 739.T	8.473	7.056	8.000
MDA-MB-157	8.189	ND	8.000
MDA-MB-436	8.114	6.255	8.000
T-47D	6.506	8.231	8.000
UACC-812	8.627	11.659	8.000
ZR-75-1	8.056	8.562	8.000

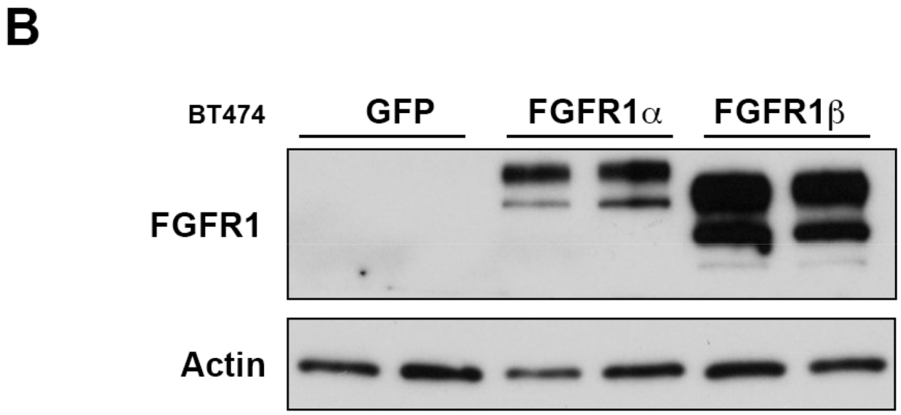

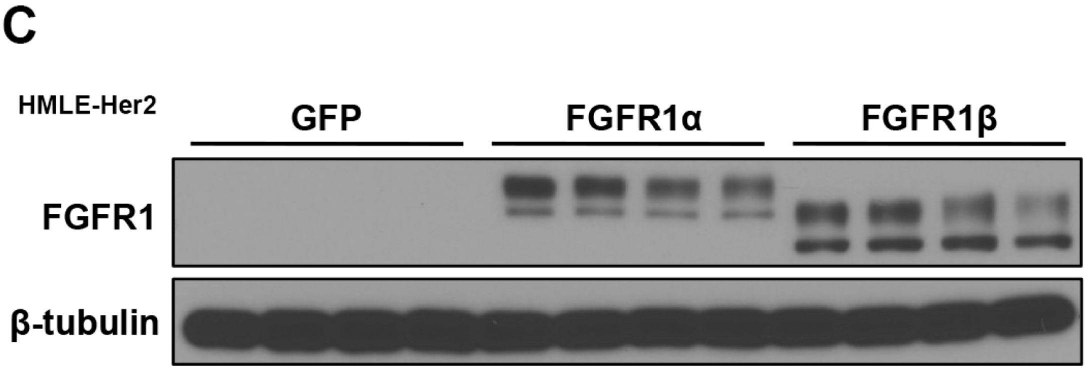

Supplementary Figure S3: FGFR1 expression correlates with lack of response to Lapatinib. A. Analysis of the CCLE database showing expression levels for FGFR1 and Her2 (ErbB2) and the corresponding IC50 values for Lapatinib in the indicated cell lines. **B.** The BT474 model of Her2 BC was transduced with viral particles encoding full length FGFR-iiic-α (FGFR1α) or the truncated -iiic isoform that lacks the outermost Ig domain (FGFR1β). Protein expression of these constructs was verified by immunoblot. **C.** Her2 transformed HMLE cells were similarly transduced with FGFR1α or FGFR1β and analyzed by immunobot. In both panels B and C β-tubulin served as a loading control.

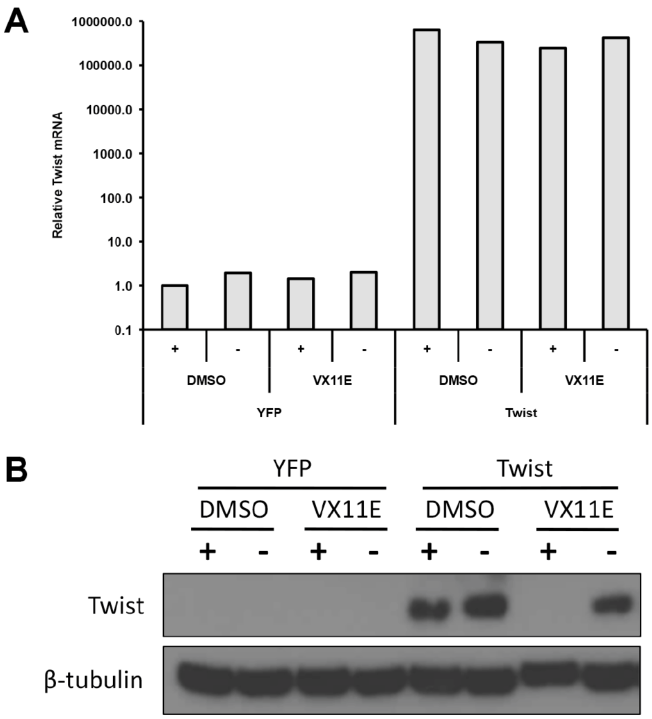

Supplementary Figure S4: Twist protein is transiently destabilized by inhibition Erk1/2. Normal murine mammary gland (NMuMG) cells constructed to overexpress Twist or YFP as a control were grown for 2 weeks in the presence of the direct ERK2 inhibitor VX11E (1μM), then cultured for one additional week either with (+) or without (-) VX11E. These cells were analyzed for **A.** Twist mRNA by RT-PCR, normalized to GAPDH, and **B.** Twist protein by immunoblot, β-tubulin served as a loading control.

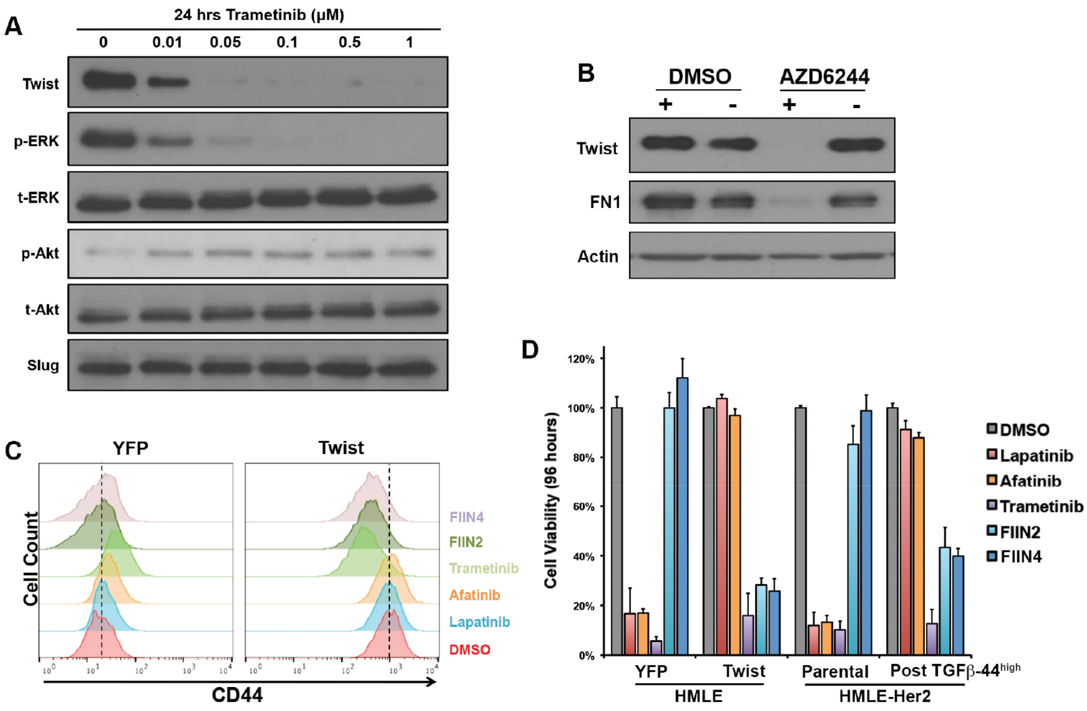

Supplementary Figure S5: Twist protein is destabilized by inhibition of FGFR:Erk1/2 signaling. A. HMLE cells constructed to stably overexpress Twist were treated with the indicated concentrations of the allosteric Mek1/2 inhibitor Trametinib for 24 hours. These cells were subsequently analyzed for expression of Twist and phosphorylation of Erk1/2 by immunoblot. Total Erk1/2 served as a loading control. Phosphorylation of Akt and expression of Slug were also analyzed to demonstrate the specificity of Trametinib for the Mek:Erk pathway and the specificity of this event for destabilization of Twist. **B.** Twist expressing HMLE cells were treated with the MEK1/2 inhibitor AZD6244 (1 µM) for 4 weeks and then split into two groups, one with continued AZD6244 treatment (+) and one without (-) and cultured for an additional week. At the completion of this period cells were assessed by immunoblot for expression of Twist and fibronectin (FN1). Actin served as a loading control. **C.** Control (YFP) and Twist overexpressing HMLE cells were treated with 1 µM of the indicated inhibitors for 96 hours the remaining cells were analyzed for CD44 expression by flow cytometry. The mean fluorescence intensity for CD44 in the DMSO treated condition for each cell line is indicated by the dashed line. **D.** Nontransformed control (YFP) or Twist overexpressing (Twist) HMLE cells and their Her2 transformed counterparts (HMLE-Her2) that were left untreated (Parental) or stimulated with TGF-β and sorted for high level expression of CD44 (Post TGF-β-44^{high}) were treated with 1 µM of the indicated inhibitors for 96 hours and subsequently analyzed for cell viability.

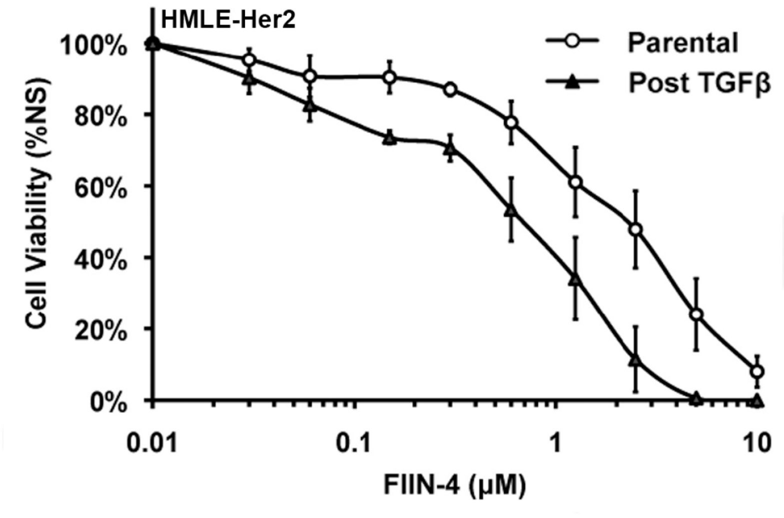

Supplementary Figure S6: Cells driven through EMT by TGF-β are more sensitive to covalent inhibition of FGFR. Her2 transformed Parental and TGF-β stimulated and recovered (post-TGFβ) HMLE cells were treated with the indicated concentrations of the covalent FGFR inhibitor FIIN4 for 96 hours and assayed for cell viability. Data are normalized to untreated cells and are the mean ± SE of at three independent experiments completed in triplicate.

Supplementary Table S1: Nanostring Tumor Progression and Metastasis Gene panel. All values are fold regulation of the indicated genes normalized to Parental HMLE-Her2 cells set to 1

See Supplementary File 1

Supplementary Table S2: The above table lists the sequence, gene target and application of all oligos used in the current study

Target	Application	Sequence (5' to 3')
hGAPDH	Real Time PCR-Sense	5'-TGCACCACCAACTGCTTAGC
hGAPDH	Real Time PCR-Antisense	5'-GGCATGGACTGTGGTCATGAG
Twist1	Real Time PCR-Sense	5'-TTCTCGGTCTGGAGGATGGA
Twist1	Real Time PCR-Antisense	5'-CTGTCCATTTTCTCCTTCTCTGG
hFGF2	Real Time PCR-Sense	5'-GCAAAAACGGGGGCTTCTTC
hFGF2	Real Time PCR-Antisense	5'-AACGGTTAGCACACACTCCT
hFGFR1	Real Time PCR-Sense	5'-CGCCCCTGTACCTGGAGATCATCA
hFGFR1	Real Time PCR-Antisense	5'-TTGGTACCACTCTTCATCTT
hFGFR2	Real Time PCR-Sense	5'-GCCTGGAAGAGAAAAGGAGATTAC
hFGFR2	Real Time PCR-Antisense	5'-GGATGACTGTTACCACCATACA
hFGFR1-iiib	RT-PCR-Sense	5'-CGGGAATTAATAGCTCGGATGC
hFGFR1-iiib/iiic	RT-PCR-Antisense	5'-TTGGTACCACTCTTCATCTT
hFGFR1-iiic	RT-PCR-Sense	5'-GGAGTTAATACCACCGACAAA
hFGFR2-iiib	RT-PCR-Sense	5'-CACTCGGGGATAAATAGCTCC
FGFR2-iiic	RT-PCR-Sense	5'-CGGTGTTAACACCACGGAC
FGFR2-iiib/iiic	RT-PCR-Antisense	5'-GGATGACTGTTACCACCATACA
FGFR1-α/β	RT-PCR-Sense	5'-TTCTGGGCTGTGCTGGTCAC
FGFR1-α/β	RT-PCR-Antisense	5'-GCGAACCTTGTAGCCTCCAA
hFGFR2-α/β	RT-PCR-Sense	5'-TTCATCTGCCTGGTCTTGGT
hFGFR2-α/β	RT-PCR-Antisense	5'-AATAAGGCTCCAGTGCTGGTTTC
hFGFR3	Real Time PCR-Sense	5'-CTCGCGCTCTGCGTGGCCGT
hFGFR3	Real Time PCR-Antisense	5'-TTCTTGTCCATCCGCTCGGG
hFGFR4	Real Time PCR-Sense	5'-GATGGACAGGCCTTTCATGGG
hFGFR4	Real Time PCR-Antisense	5'-TGCTGCGGTCCATGTGGGGTCCTC

Supplementary Table S3: The above table lists the vendor, catalogue number and dilution factor of all the antibodies used in the current study

Antibody	Dilution	Supplier (Catalogue #)
E-Cadherin	1:2000	BD Biosciences (610182)
Her2	1:1000	Cell Signaling Technologies (4290)
Vimentin	1:1000	BD Biosciences (550513)
CD44-FITC	1:100	Bio Legend (338804)
CD24-PercP	1:100	Bio Legend (311113)
β-Tubulin	1:1000	DSHB (E7)
Twist	1:100	Abcam (ab50887)
Phospho-Akt	1:1000	Cell Signaling Technologies (4060)
Total-Akt	1:1000	Cell Signaling Technologies (9272)
Total-Erk1/2	1:2000	Cell Signaling Technologies (4695)
Slug	1:1000	Cell Signaling Technologies (9585)
β-actin	1:1000	Santa Cruz Biotechnologies (sc-1616)
N-cadherin	1:1000	BD Biosciences (610920)
Fibronectin	1:1000	BD Biosciences (610078)
FGFR1	1:1000	Cell Signaling Technologies (9740)
ITGB3	1:1000	Cell Signaling Technologies (4702)
Phospho-Erk1/2	1:2000	Cell Signaling Technologies (9101)
